# Experimental Investigation on the Morphology and Adhesion Mechanism of Leech Posterior Suckers

**DOI:** 10.1371/journal.pone.0140776

**Published:** 2015-11-04

**Authors:** Huashan Feng, Ningli Chai, Wenhao Dong

**Affiliations:** 1 School of Mechanical Engineering, Northwestern Polytechnical University, Xi’an, China; 2 Department of Gastroenterology, Chinese Peoples Liberation Army General Hospital, Beijing, China; University of Zurich, SWITZERLAND

## Abstract

The posterior sucker of a leech represents a fascinating natural system that allows the leech to adhere to different terrains and substrates. However, the mechanism of adhesion and desorption has not yet to be elucidated. In order to better understand how the adhesion is performed, we analyzed the surface structure, adsorption movements, the muscles’ distribution, physical characteristics, and the adsorption force of the leech posterior suckers by experimental investigation. Three conclusions can be drawn based on the obtained experimental results. First, the adhesion by the posterior sucker is wet adhesion, because the surface of the posterior sucker is smooth and the sealing can only be achieved on wet surfaces. Second, the deformation texture, consisting of soft collagen tissues and highly ductile epidermal tissues, plays a key role in adhering to rough surfaces. Finally, the adhesion and desorption is achieved by the synergetic operation of six muscle fibers working in different directions. Concrete saying, directional deformation of the collagen/epithermal interface driven by spatially-distributed muscle fibers facilitates the excretion of fluids in the sucker venter, thus allowing liquid sealing. Furthermore, we found that the adhesion strength is directly related to the size of the contact surface which is generated and affected by the sucker deformation. Such an underlying physical mechanism offers potential cues for developing innovative bio-inspired artificial adhesion systems.

## Introduction

Recently, increasing efforts have been made to investigate the special role of biological adhesion to understand how it works effectively on complicated surfaces, and therefore to mimic its function by developing an artificial structure. For instance, it has been reported that an array of artificial bristles inspired by the gecko feet setae is able to adhere to smooth surfaces based on van der Waals forces [1]. Such a structure is made of organic polymers is another example imitates the contact surface of the spider feet which is characteristic of a flexible array of tiny thorns and compliant suspension, with the aim to climbing rough surfaces [2]. The mouthparts of a tapeworm was also studied, and accordingly an artificial hook was developed to mimic its surface and drive providing a piston driven by spiral ionic-polymer metal composite (IPMC) to fix on gastrointestinal tracts [3].

As a specialized organ, suckers are an adhesion tool widely observed in creatures, such as parasitic worms [4], cephalopods [5], fish [6], bats [7]. More specifically, these creatures possess disk-like muscle tissues that enable adhesion to host or substrate. Nevertheless, the negative pressure adhesion used by these creatures differs from the conventional negative pressure adhesion in that the adhesion is achieved by pumps or other assistant tools to extrude the air. Therefore, negative pressure is formed by the muscle movements and the specific morphology of the suckers. Additionally, the adhesion is non-destructive and suitable for both wet and rough surfaces. Compared to the conventional mechanical negative pressure adhesion, this methodology shows great potential for industrial applications as it is environmentally friendly, easy-to-use, and effective. Therefore, studies on biological suckers aiming to facilitate the development of biomimetic suckers are of great importance.

As a typical annelid with suckers, leeches are widely spread in nature. These creatures live on blood from animals. Previous studies on leeches focused on medical applications, such as there-attachment of body parts, reconstructive and plastic surgeries [8], and osteoarthritis treatments [9][10]. Each leech has two suckers: the small one on the head is the mouthpart, which enables biting on epidermal tissues; the other one on the tail (3-7mm in diameter), which allows adhesion on contact surfaces and leverage. Due to its complicated structure (mouthparts included), anterior suckers have been widely investigated [11] [12], while study of the working mechanisms of posterior suckers are still at an early stage, with few reports on this topic published in the past century. In comparsion with suckers of other creatures (e.g. octopus, fish, and bat), the posterior suckers of a leech is characteristic with a reduced size and a simple adhesion structure. The synergetic operation of its muscles and epithermal tissues enables adhesion on and desorption from wet surfaces. For these reasons, the posterior suckers of a leech shows great potential in the field of biomimetic suckers. However, the mechanism of adhesion and desorption was not well understood. Herein, we present a study of the fundamental mechanisms of adhesion and desorption of a leech posterior sucker by investigating its structure, conformation, and movements.

## Materials and Methods

Among various leeches, the *Whitmaniapigra* (*Whitman*, *1884*) was selected and used as the target in this study. *Whitmaniapigra* is one species of the five genera of Annelids that includes *Whitmania*, *Blanchard*, *Haemopidae*, *Arhynchobdellida*, and *Hirudinea*. The *Whitmaniapigra* is relatively large and the movements of its posterior suckers can be easily observed. Moreover, this creature is easily accessible as it has been commercially cultured. Between April and August 2014 a total of 78 individuals were obtained from commercial leech wholesaler. All specimens were fed and collected by the Qin Hetang Big Pharmacy Co. Ltd. It is a traditional Chinese medicine firm that holds permits to collect and sale leeches. The firm is located in Xuzhou City (Northwest Jiangsu Province, Eastern China). Healthy leeches were hand-collected on the wholesaler within one day of capture, and then mailed to the authors within two days of arriving Xi'an (Shaanxi Province, China). In the laboratory they were initially housed in glass containers with removable glass mesh lids (20L). Water temperature was 20–23°C. The water was changed every two days to prevent it deterioration. Leeches were fed every ten days a variety of snail. When they were fully adapted to the laboratory environment, the active leeches were used in the experiments. The adult leeches used in this study were 6–13cm in length, 0.8–2cm in width, and 5–10g in weight. The study protocol was approved by the animal ethics review board of Northwestern Polytechnical University. In order to study the adhesion process, the surface structure, physical properties, muscle distribution, adsorption interaction, and force of the leech posterior suckers were investigated.

### Surface Structure

As the first step, the surface morphology of suckers was investigated through the paper. The posterior suckers cut from living leeches may shrink to a certain extent, thus resulting in wrinkles on their surface. Therefore, the suckers were cut at the narrow joints and immersed in Ringer's solution (115 mM NaCl, 4 mM KCl, 1.8 mM CaCl_2_, 10 mM TrisHCl buffer, pH = 7.4) for 10 minutes to keep the muscles relaxed. Then, the suckers were washed with normal saline and 5% sodium bicarbonate, followed by rinsing with trypsin solution and natrium cacodylicum buffer to remove the residual mucus on their surface. Finally, the samples were dehydrated with graded ethanol ranging from 10% to 100% and then incubated in 2.5% glutaraldehyde solution [12][13]. Afterwards, the suckers (3 mm in diameter) were observed using an environmental scanning electron microscope (ESEM; FEI Quanta 450) at 5°C, 7.33×10^2^ Pa and 85% relative humidity [14][15].

### Adhesion Process

The adhesive force for crawling and adhesion of leeches is provided by their posterior suckers. As the posterior suckers are relatively small and covered by the body, the fast and inconspicuous movements cannot be observed with the naked eye. For this reason, High speed digital cameras with macro lens (IDT, Y7-S2, pixel = 2 million, resolution = 1920 x 1080) were employed to capture (laterally and ventrally) the movements of suckers during the crawling of a leech on a transparent slide [16].

### Muscle Distribution

To investigate the muscles’ distribution in leech posterior suckers, paraffin sections with hematoxylin-eosin stain were prepared using the following procedure. First, sucker samples were fixed in Bouin’s solution (obtained from Sigma-Aldrich) for 12h and stored in 70% ethanol. Then, the samples were dehydrated using graded ethanol series (from 50% to 100%, 2h for each concentration) and embedded in the as-prepared paraffin. Serial-sections (4-μm-thick) cut with a microtome were stained with hematoxylin-eosin and characterized using a light microscope (Leica DM 6000B) [17].

### Texture Analysis

In order to determine physical properties of the posterior suckers (e.g., hardness, adhesiveness, elasticity, cohesiveness, adhesion, and recoverability), a texture profile analysis (TPA) using Texture Analyzer (SMS TA-XT Plus) was conducted. Ten leeches were chosen and their posterior suckers were cut as the samples. Ten leeches were chosen and their posterior suckers were cut as the samples. Herein, these 10 samples were divided into two groups: samples in Group A were treated by incubation in Ringer's solution, while samples in Group B were not treated. A Φ35 cylinder probe was used and samples were pressed twice to achieve 75% deformation [18].

### Adhesive Force

The adhesive forces of 12 leeches which differs in size were tested using a tension meter at 15–20°C. Each leech was wrapped by a piece of gauze, which was tied to the tension meter. Once the leech sucked on the slide, the tension meter was pulled away until the leech was separated from the glass. By this process, the maximum force measured by the tension meter was regarded as the adhesive force. Diameters of the suckers were measured at the back of the glass. The chosen leeches were divided into four groups based on their sucker sizes.

## Results

### Surface Structure

**Fig 1:**
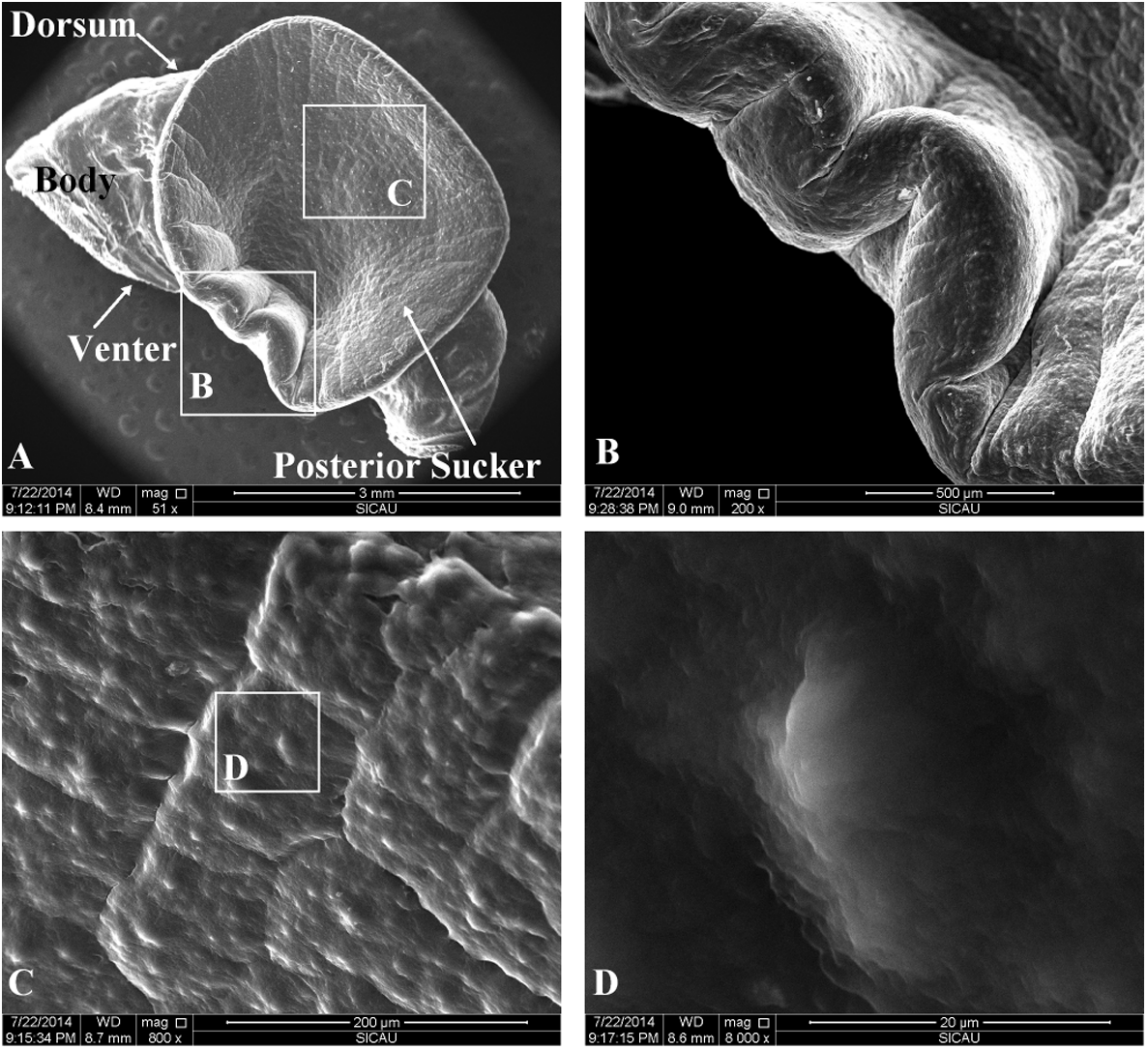
The ESEM images of a relaxed sucker surface (back view). A: the top view of a sucker sample. The sucker is relaxed, with its open mouth facing up. The body is the residual ring structures. B: a zoom-in (200x) image of the wrinkles. C and D: the 800x and 8000x images of the smooth internal surface of the sucker. As shown, the surface of the posterior sucker is extremely smooth.

The ESEM images of a relaxed sucker surface (back view) are shown in Fig 1. This sucker shows a disk-like structure, with its relaxation maximized in the radial direction (5–6 mm) and its thickness decreasing along the radius. This translucent surface shows less pigmentation than dermal tissues in other parts of the leech. The epidermal tissues on the sucker venter are in irregular polygonal sections, with tiny grooves in the boundary regions for expelling water and gas. The surface is relatively smooth, although tiny bulges were observed. Additionally, no setae or bristles was observed on the sucker.

### Adhesion Process

**Fig 2:**
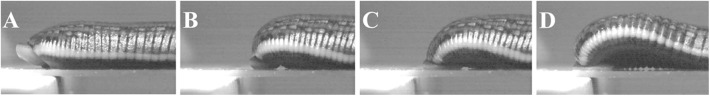
The adhesion process of the posterior sucker. A: the sucker bent down and the inner surface uncovered; B: the inner surface in contact with the substrate; C: the sucker stretched out along the radial direction and its thickness decreased; D: the ultimate morphology of the sucker (contact area is maximized while thickness is minimized).

**Fig 3:**
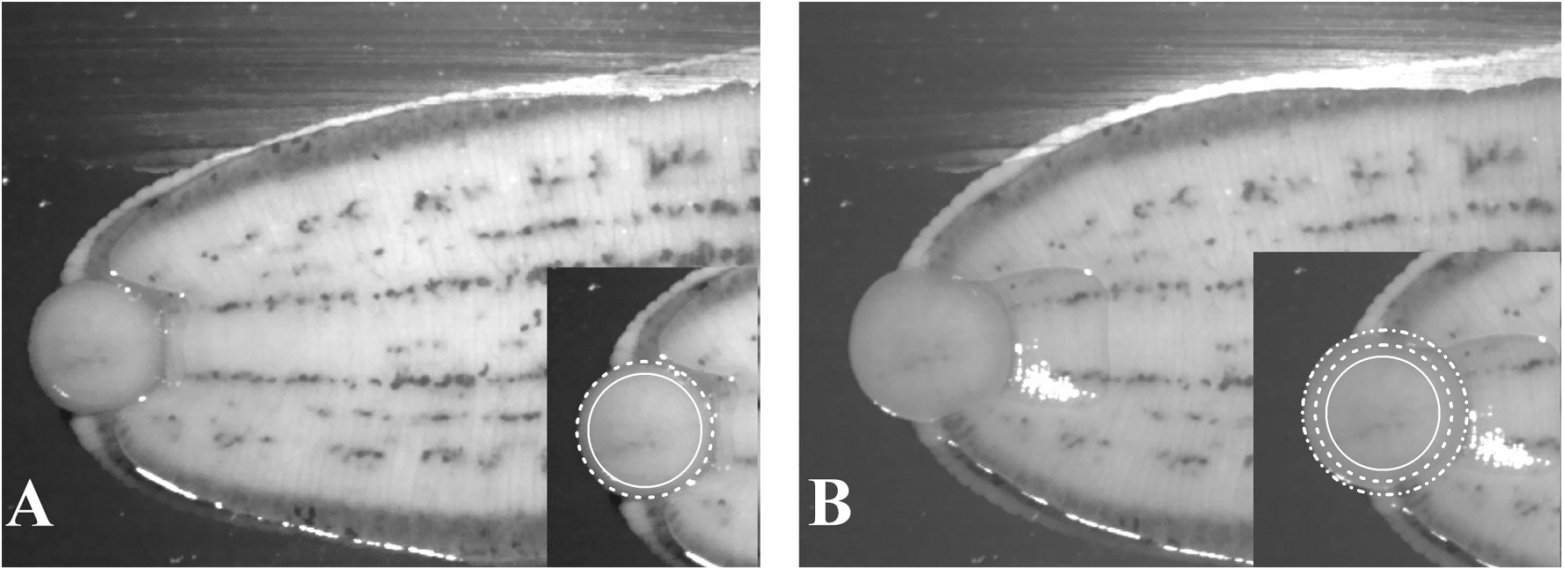
The bottom view of the suckers in the adhesion process. A: the initial contact between the suckers and the slide; the area labeled with a dashed line is the sucker shadow, while the area labeled with a solid line is the actual contact area; B: the sucker after complete adhesion; the contact area is larger than the initial area due to the stretching of the sucker.

**Fig 4:**
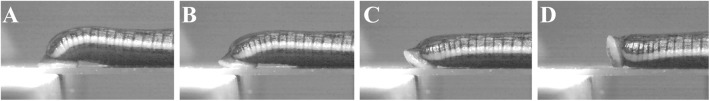
The desorption process of the posterior sucker. A: the suckers contracted starting from the edge and the contact area decreased; B: the edges of the suckers detached from the substrate; C: the sucker bent up; D: the entire detached sucker and body-driven movement.

**Fig 5:**
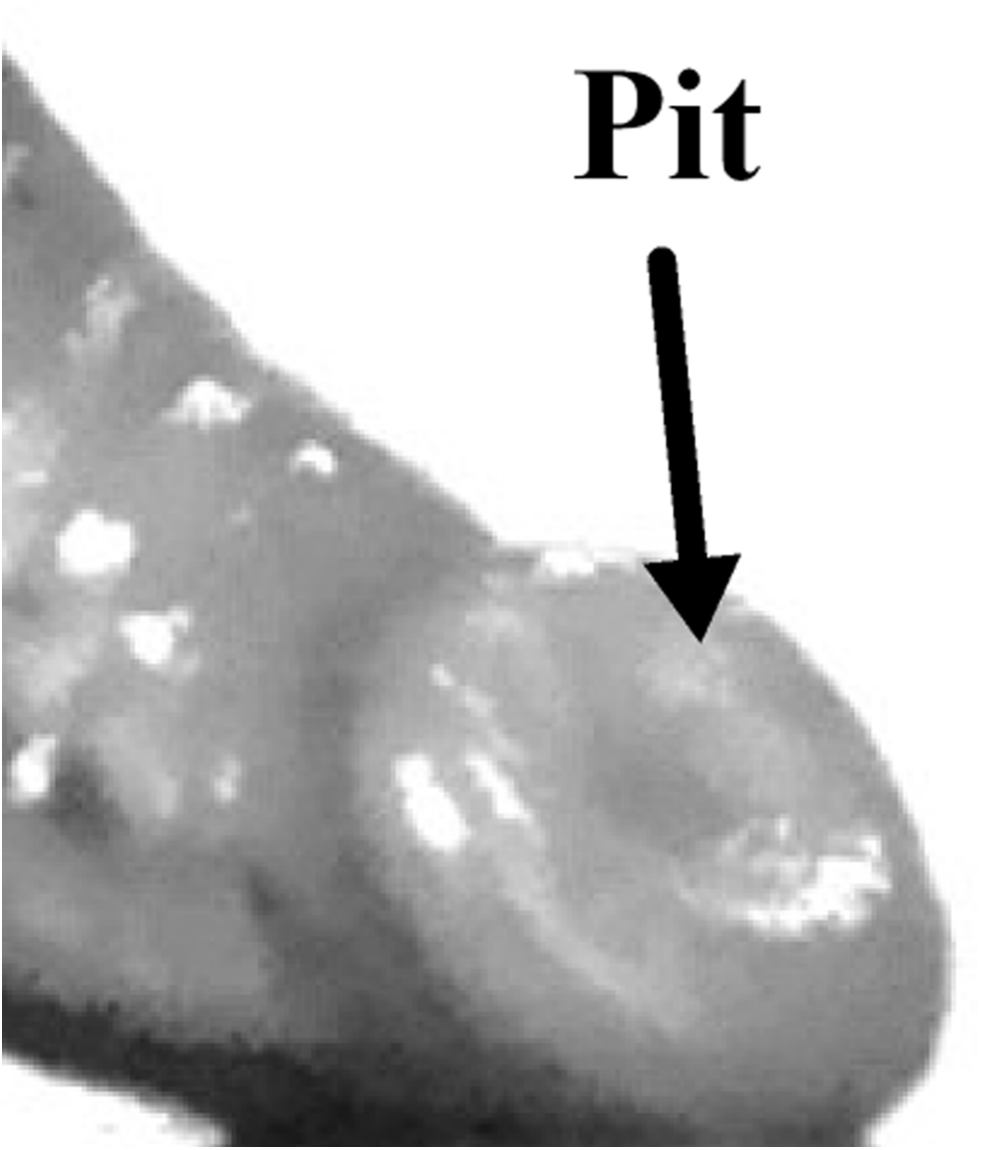
The pit was formed on the inner surface as a result of the sucker free contraction.

The adhesion process of the posterior sucker can be divided into two stages: adhesion and desorption. In the adhesion stage (Fig 2), the body contracted followed by the sucker bending inwards, with its internal surface bending outwards, as shown in Fig 2A. Upon contacting the substrate (Fig 2B), the suckers stretched along the radial direction, while the thickness decreased (Fig 2C). Ultimately, the suckers were spread out on the substrate to the fullest extent (Fig 2D). Fig 3 shows the bottom view of the suckers in the adhesion process. Fig 3A shows the initial contact between the suckers and the slide. The area labeled with a dashed line is the projection of sucker shape, while the area labeled with a solid line is the actual contact area. With Fig 3A, it can be concluded that the actual contact area is smaller than the projective area due to the outward bending sucker. Fig 3B shows the sucker after completing adhesion. At this stage, the contact area is enlarged as compared to the initial area. The additional area is the stretching area of the sucker. This is confirmed by the reflection points at the edges of the sucker. In the adhesion process, the inner surface bent towards the central part of the sucker. In the desorption process (Fig 4), the suckers contracted, starting from the edge (Fig 4A), and the contact surface area decreased. Concretely speaking, the edge of the suckers detached from the substrate (Fig 4B), followed by detachment of the entire sucker (bending up) with its movement driven by body movements (Fig 4C). At the end, the suckers are completely detached and moved with the body (Fig 4D). Pit is formed on the inner surface of the sucker as a result of contraction. The pit can only be observed in cases where “free contractions” are allowed (see Fig 5). “Free contractions” refers to the contractions of the suckers when the leech is scared or stimulated by the external environments. In this case, the suckers’ inner and outer surfaces contract severely inward, thus resulting in a spherical body. As a consequence, the pit in the inner surface disappears, and instead the outward-protruding appearance is generated.

### Muscle Distribution

**Fig 6:**
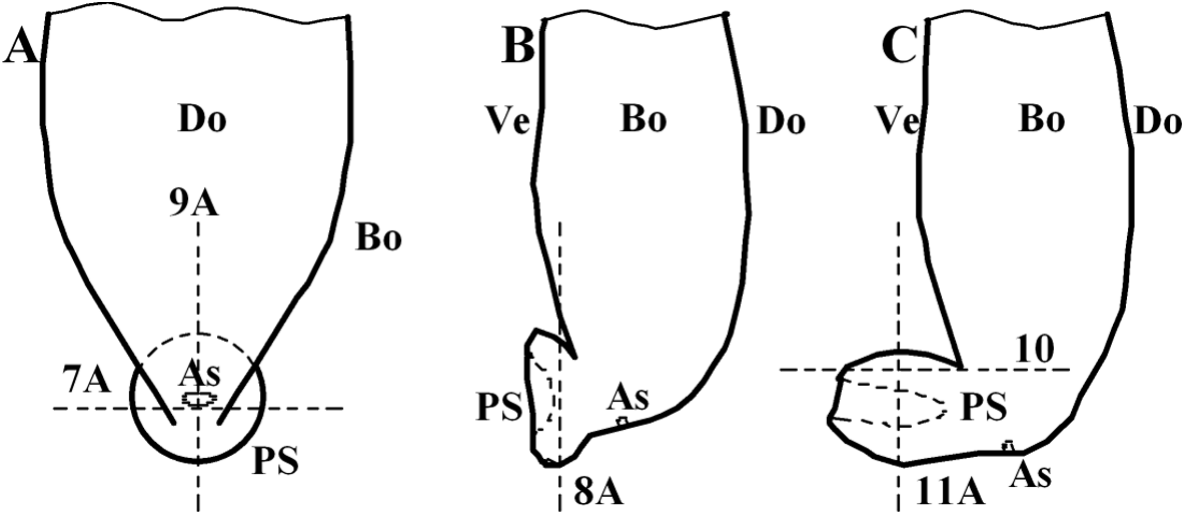
Illustrations of the sectioning planes across the body of suckers. A: the ventral view of the posterior sucker; B: the lateral view of the contracted posterior sucker; C: the lateral view of the relaxed posterior sucker. Bo: Body of the leech, PS: Posterior sucker, As: Anus, Do: Dorsum, Ve: Venter. 7A, 8A, 9A, 10, 11A: Fig 7A, Fig 8A, Fig 9A, Fig 10 and Fig 11A.

**Fig 7:**
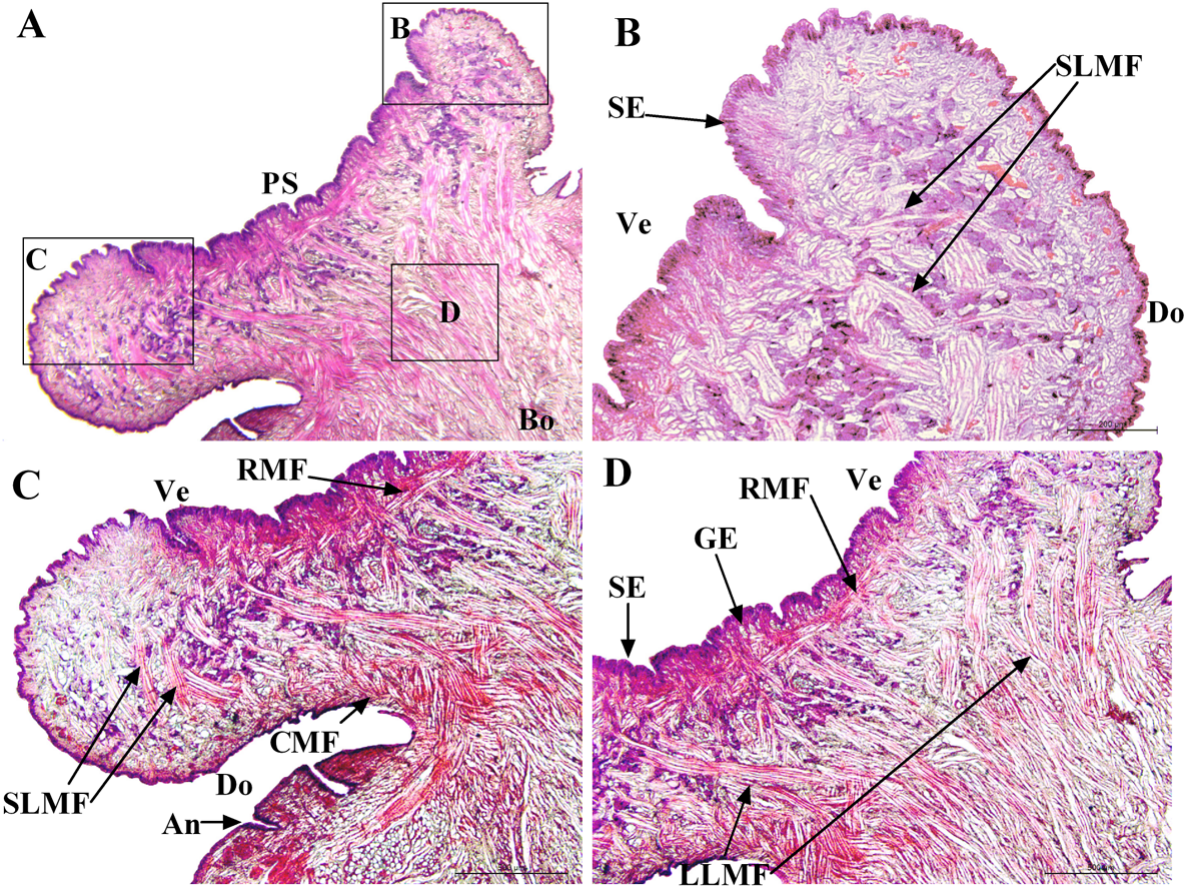
The coronal sections of the contracted posterior sucker. Four muscle fibers can be observed: long longitudinal muscular fiber (LLMF), short longitudinal muscular fiber (SLMF), circular muscular fiber (CMF), and radial muscular fiber (RMF). Minor muscle fibers can also be observed in the area beneath epidermal tissues of the sucker and body joints. SE: stratified epithelium, An: annuli of the body, GE: glandular elements. The scale bars = 200 μm in Fig 7B and 500 μm in Fig 7C and 7D.

**Fig 8:**
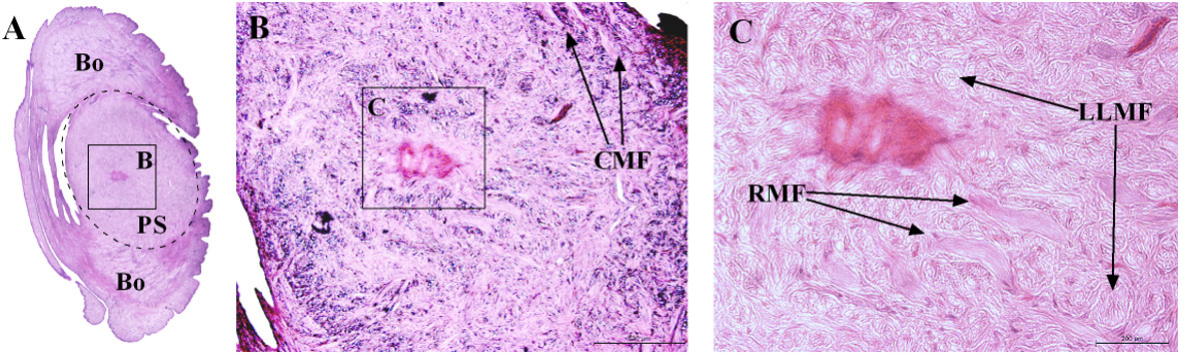
The cross sections of the contracted posterior sucker. The area labeled by the dashed line is the sucker, while the outer part is the body. Starting from the central part of the sucker, RMF are distributed radially. LMF are shown in a cross sectional view. CMF can be observed at the edge of the sucker. Scale bars = 500 μm in Fig 8B and 200 μm in Fig 8C.

**Fig 9:**
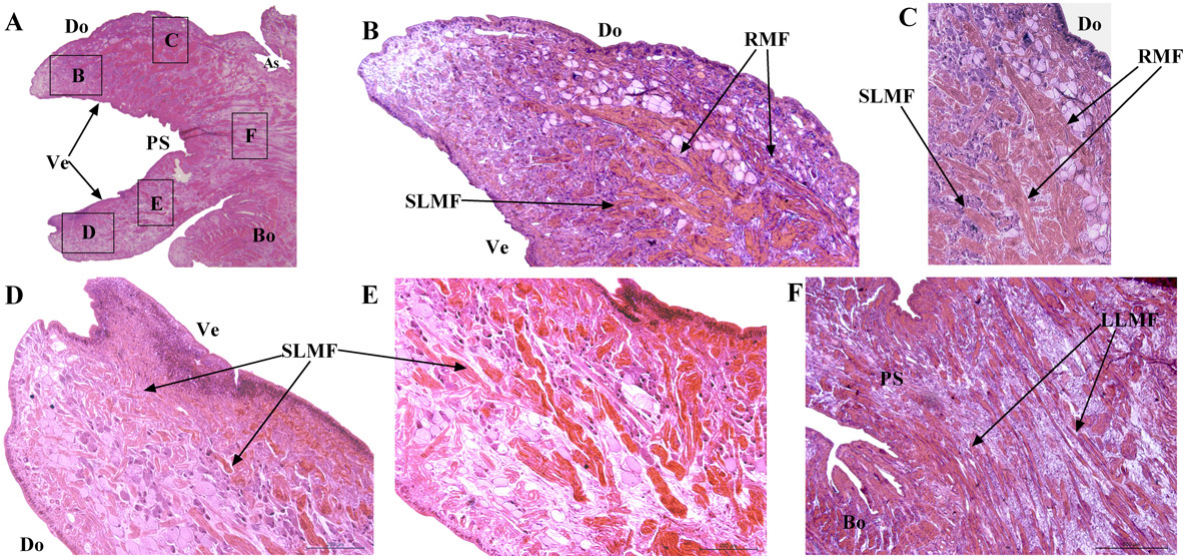
The coronal sections of the relaxed posterior sucker. The SLMFs located between the venter and dorsum of the sucker are significantly shorter than the LLMF. These relaxed LLMF are slightly longer than contracted ones. Radial muscle fibers located beneath the dorsal epidermal tissues of the sucker are observed; these fibers are in relaxed state. Scale bars = 200 μm in Fig 9B, 9C, 9D and 9E and 500 μm in Fig 9F.

**Fig 10:**
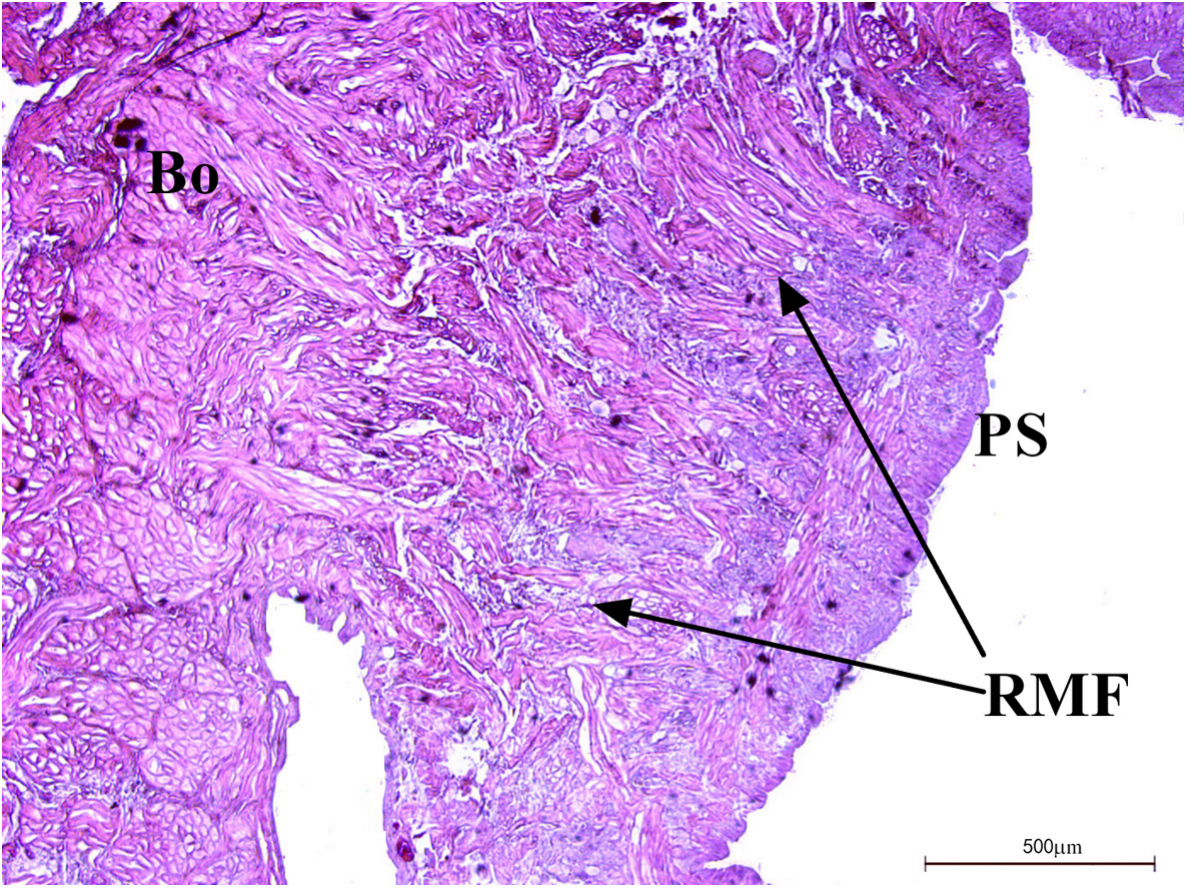
The transverse sections of relaxed posterior sucker. Radial muscle tissues are also distributed along the dorsal epidermal tissues of the sucker. Scale bars = 500μm.

**Fig 11:**
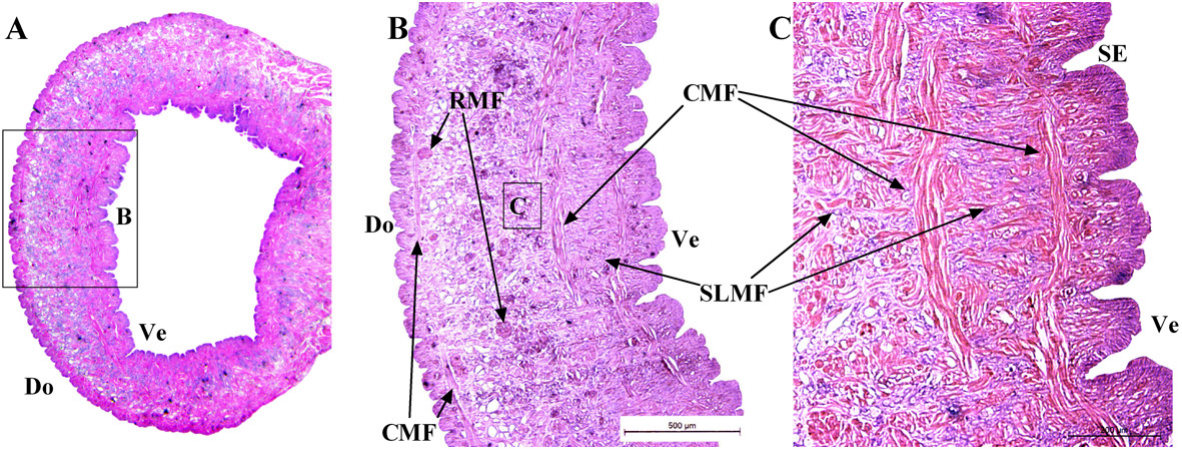
The ring-shaped coronal section of a relaxed sucker. The other two CMF that are regularly distributed can also be observed on the dorsum and the venter of the sucker.

The distribution of muscles in the posterior sucker is more complicated than that in the body [19]. Therefore, three samples are treated with muscle relaxation methods before incubation in the fixative solution, while the other two are not treated. Slices of the vertical plane, coronal section and cross section of each sample are obtained, and illustrations of the sectioning planes across the body of suckers shown in Fig 6. Figs 7A and 8A show the coronal and cross sections of the contracted posterior sucker, while Figs 9A, 10A and 11A show the vertical plane, coronal section, and cross section slices of the relaxed posterior sucker.

The posterior sucker is covered by a single stratified epithelium (SE) with a thin cuticle supported by numerous muscular fibers and glandular elements (GE, see Figs 8 and 11). The glandular elements serve as linkers between muscle fibers in the stratified epithelium, resulting in a huge complex. The gland opening is on the adhesion surface of the sucker venter and secretions can be excreted from the opening. The internal part of the sucker consists of interlaced smooth muscle fibers, with intervals filled with connective tissues. With H&E staining, pink collagen tissue (CT), purple elastic fibers, and colorless lattice fibers could be observed.

In Fig 7A, four muscle fibers, including a long longitudinal muscular fiber (LLMF), a short longitudinal muscular fiber (SLMF), a circular muscular fiber (CMF), and a radial muscular fiber (RMF), can be observed. In Fig 7D, the LLMF consisted of long connective tissues that are distributed from the last body septum to the area beneath the stratified epithelium of the ventral sucker. These fibers are perpendicular to the epidermal tissues of the sucker venter and in a contracted state. Illustrated in Fig 7B and 7C, the SLMF consisted of short connective tissues that are distributed between the ventral and dorsal epidermal tissues. These fibers are perpendicular to both parts and in a relaxed state. In Fig 7C, the CMF are located beneath the dorsum that is close to the sucker root. In Fig 7C ans 7D, the RMF are located beneath the stratified epithelium of the sucker venter; these fibers are in a contracted state. Additionally, muscle fibers can also be observed in the area beneath the epidermal tissues of the sucker and body joints. As shown in Fig 8A, the RMF are in a radial distribution starting from the central part of the sucker. These fibers are in a contracted state (see Fig 8C). Inevitably, the slice cut contains body tissues; the coronal section of the sucker is labeled with a dashed line to distinguish it from other tissues. For the LLMF sample, only the cross section slice is shown. Fig 8B shows the distribution of CMF at the sucker edge.

In Fig 9A, the size of the relaxed sucker was significantly larger than that of the contracted sucker in terms of both area and volume. Indeed, the diameter of a relaxed sucker could be twice that of a contracted one. According to the image of the vertical plane slice, SLMF located between the venter and dorsum of the sucker were significantly shorter (see Fig 9B and 9D). The relaxed LLMF were slightly longer than the contracted ones (see Fig 9F). Besides the four muscle fibers mentioned above, three other fibers were also observed. The first one was the radial muscle fibers located beneath the dorsal epidermal tissues of the sucker; these fibers were in a relaxed state (see Fig 9B and 9C). Radial muscle tissues were also radially distributed along the dorsal epidermal tissues of the sucker, as shown in Fig 10. The coronal section of the relaxed sucker is ring-shaped (see Fig 11A). The other two CMF were regularly distributed and could also be observed on the sucker dorsum and venter (see Fig 11B and 11C). The dorsum fibers (DoCMF) are single-layered, while the venter fibers (VeCMF) are double-layered or multi-layered. The diameter of the VeCMF was larger than that of the DoCMF. Additionally, relaxed SLMF (length = 10 μm) that were located between and perpendicular to the ventral and dorsal epidermal tissues of the sucker were also observed.

### Texture Analysis

**Fig 12:**
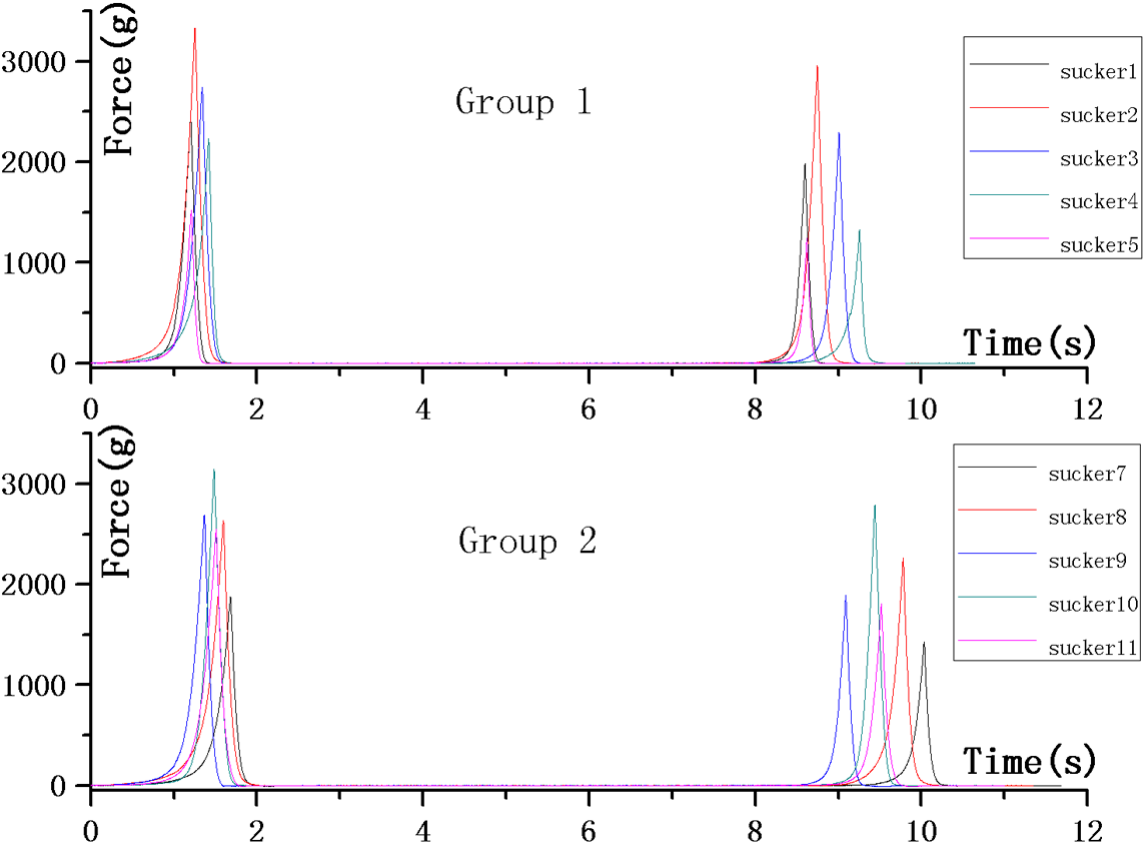
The results of texture analysis revealed the structural characteristics of leech sucker tissues.

The H&E stain analysis showed that the posterior sucker of a leech consists of not only muscle tissues but also connective tissues made of collagen. The collagen tissues are highly durable, while the elastic tissues are highly elastic. The results of a texture analysis (shown in Fig 12) revealed the structural characteristics of these tissues. Table 1 shows the average values and variances of all sucker samples. The samples of Group One are obtained from living creatures without any treatment, while the samples of Group Two are treated with muscle relaxation methods. Because no significant difference is observed for the curves of these two groups, results of all samples analyzed as a whole.

**Table 1:** The average values and variances of all sucker samples (Data in S2 Dataset).

Parameters	Unit	Group 1		Group 2		Overall	
		Mean	Variance	Mean	Variance	Mean	Variance
Hardness	g	2452.562	665.985	2579.372	454.613	2515.967	541.709
Adhesiveness	g∙sec	- 4.730	0.649	- 4.537	1.156	4.633	0.890
Elasticity		0.592	0.064	0.592	0.051	0.592	0.054
Cohesiveness		0.615	0.107	0.603	0.113	0.609	0.104
Gumminess		1545.475	635.433	1578.050	529.798	1561.762	551.815
Recovery		0.561	0.111	0.556	0.127	0.559	0.113

The hardness index in the texture analysis indicated the force applied for maximum deformation of the sucker. The sucker is highly deformable. The elasticity, recovery, and cohesiveness indices indicated the recovery capability of the sucker. The results revealed that the recovery index is greater than 50%, indicating good durability and recovery capabilities. The adhesiveness results revealed that the adhesion of the sucker itself is relatively low, indicating that physical adhesion is not the key working principle of suckers. On the other hand, the collagen tissues in the suckers showed high adhesiveness; this is in agreement with the observation of the slices. Therefore, it can be concluded that the collagen tissues play a key role in the deformation and recovery of the sucker.

### Adhesive Force

**Fig 13:**
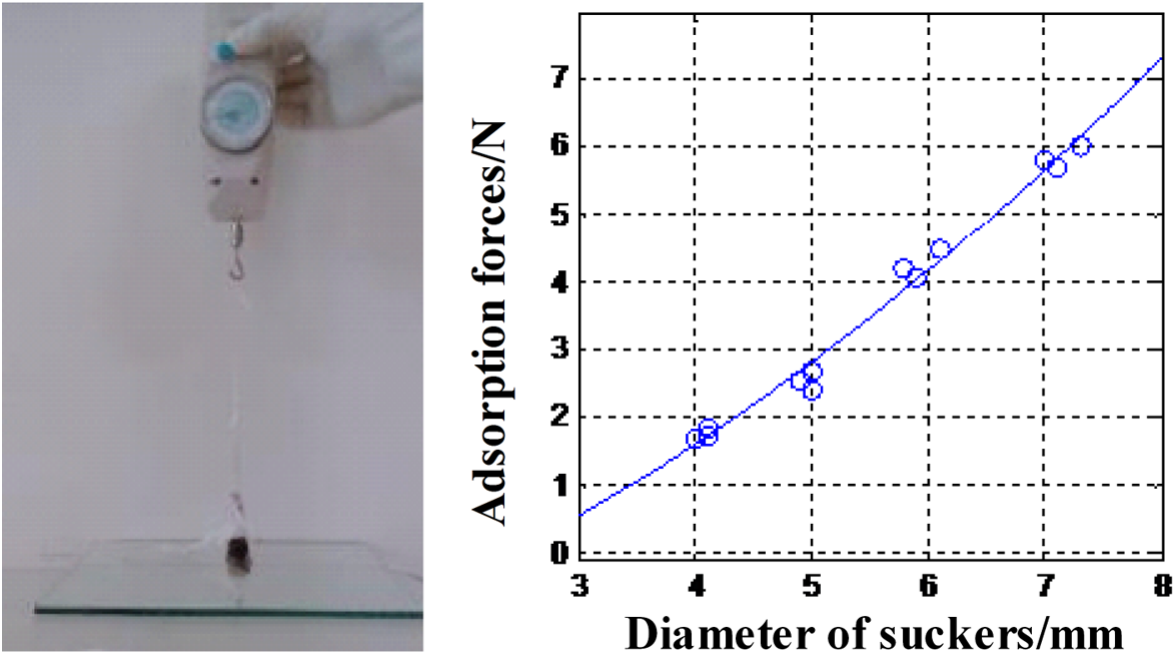
The adsorption forces of different suckers (Data in S1 Dataset).

In Fig 13, instead of the theoretical quadratic function, the relationship between the adhesive force and the diameter of the sucker is approximately linear. The adhesive force generated by a sucker of 6 mm in diameter is up to 4.5N, whereas the atmospheric pressure on an area of 28 mm2 is around 2.85 N. Therefore, the negative pressure created by the sucker is calculated to be 58 kPa, which is about 0.57 times atmospheric pressure. Furthermore, it is clear that the adhesive force provided by the sucker increased with its contact area.

## Discussion

Based on the obtained experimental results, it can be concluded that adhesion and desorption of the posterior sucker of a leech rely on three parameters.

The first parameter is the properties of epidermal tissues. Leeches have no setae or bristles on their epidermal tissues, and these tissues are not adhesive. Indeed, adhesion is achieved by the fixative interaction between epidermal tissues of the sucker and the substrate. This interaction is realized by the moisture on the sucker/substrate interface, namely wet adhesion. The moisture is provided by secretion from the anus or glands on dorsal tissues. For this reason, long distance crawling leads to dehydration and body shrinkage for leeches. The grooves on the epidermal tissues enable a uniform distribution of secretions on the whole surface of the sucker for wet sealing to achieve negative pressure. Therefore, the sucker is pressed on the substrate by the atmosphere. On the other hand, surface roughness is increased by bulges on the sucker so that skidding is minimized.

The second parameter is the physical properties of the sucker. The leech sucker is a complex consisting of epidermal tissues, smooth muscle tissues, and collagen tissues. The sucker is relatively soft and highly deformable. This can be attributed to the deformable collagen tissues and the driving force for deformation provided by smooth muscle tissues. Meanwhile, the epidermal tissues define the outline of the sucker after deformation. This highly deformable complex, which consists of soft filler and integument, shows great adaptability for different surfaces. The working principle of this complexity is similar to that of the universal jamming gripper, which is capable of capturing various irregularly shaped objects [20].

**Fig 14:**
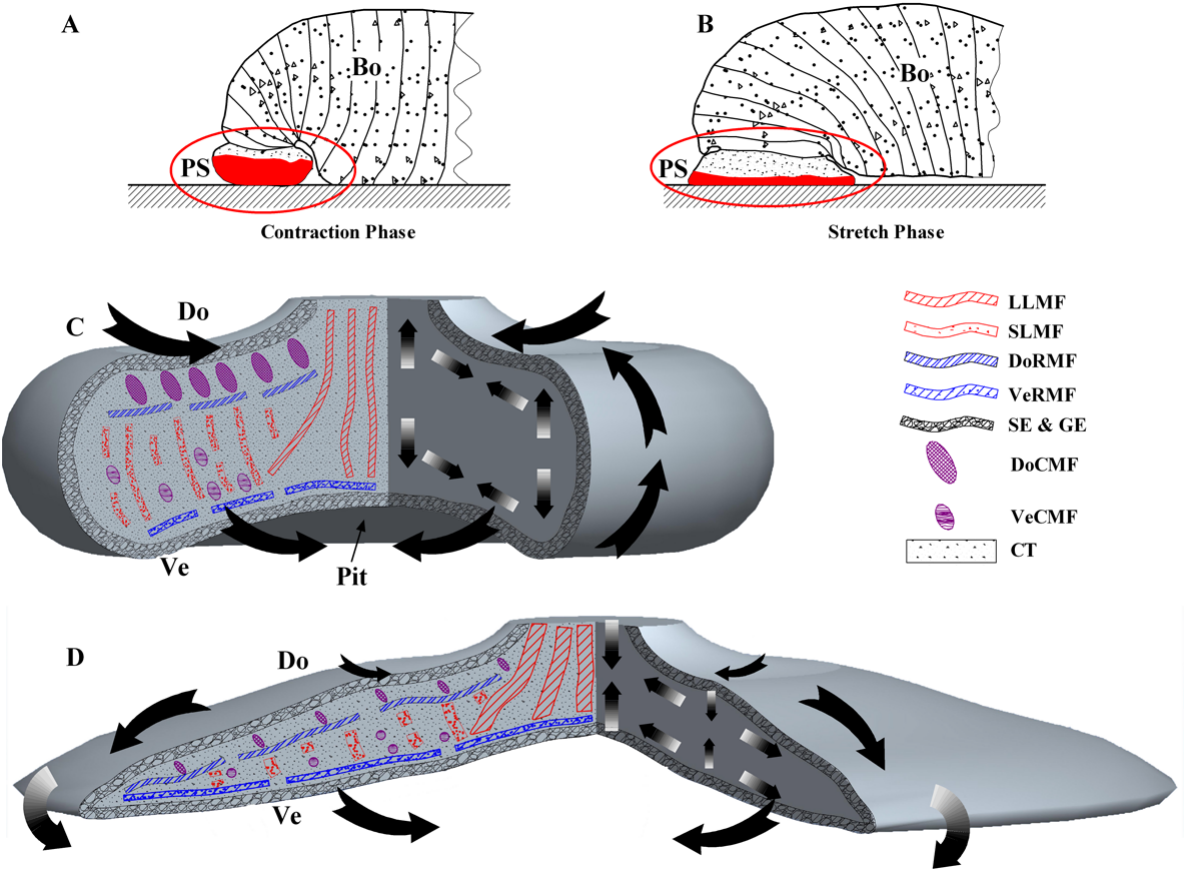
Schematic view, in two phases, of the adhesion mechanism proposed for the leech posterior sucker. In the desorption process, the sucker contracted to reduce the contact area and negative pressure in the contact region (Fig 14C). Specifically, the DoCMF and DoRMF contracted so that the dorsum was reduced and the edges bent up to allow air in. In contrast, the VeCMF and VeRMF contracted so that the sucker venter shrank; the LLMF and SLMF were forced to relax due to the deformation caused by collagen movement, resulting in thickening of the sucker.

The third parameter is the distribution of muscle tissues and the synergistic interactions between these tissues. In this study, the muscle tissues were categorized as dorsum circular muscular fiber (DoCMF), venter circular muscular fiber (VeCMF), dorsum radial muscular fiber (DoRMF), and venter radial muscular fiber (VeRMF). In the posterior suckers of leeches, six different categories of muscle tissues were observed, illustrated in Fig 14. Adhesion and desorption are associated with two processes: contraction (Fig 14A) and relaxation (Fig 14B). Adhesion is realized by the sucker relaxation, while desorption is realized by the sucker contraction.

In the adhesion process, the sucker relaxed to increase the contact area and achieve negative pressure in the contact region (Fig 14D). To achieve it, the SLMF contracts, the collagen tissues are pushed outwards and the ventral epidermal wrinkles disappears. The fluids in the contact region is expelled via the surface grooves and sealing (negative pressure) achieved. The DoRMF and VeRMF stretch and the sucker surface enlarge in a radial direction. The DoCMF is single-layered, while the VeCMF is multi-layered and shows larger diameters. Therefore, the pulling force by DoCMF is relatively weak and stretching of these fibers is observed prior to that of VeCMF. As a result, the sucker edges bends downwards and the contact surface increases gradually. Additionally, contraction of LLMF leads to a pit in the central part of the sucker, thus enhancing the adhesive force.

## Conclusions

First, the adhesion of leeches is a wet adhesion, as their sucker venter is relatively smooth and the sealing can only be achieved with wet surfaces. Second, the adhesion and desorption of the posterior suckers are closely related to the sucker morphology, including the distributions of epidermal tissues, collagen tissues, and muscle fibers. The structures implicated in deformation consist of soft collage tissues and highly ductile epidermal tissues, enabling the adhesion of suckers on rough surfaces. The sucker adhesion is also a negative pressure adhesion. The adhesion or desorption process requires coordinated movements from six muscle fibers in different directions.

The adhesion mechanism of posterior sucker is derived from the discussion above. First the widely distributed muscle fibers drive the direction of deformation in the epidermal and collagen tissues. As a consequence, the directional deformation is generated on the contact surface. This results in the discharge of the air and liquid in the belly and the formation of the liquid sealing.

The contact surface after deformation is a determining factor of the adhesive force. Future works will explore the possibility of biomimetic suckers with novel materials and morphologies, with the intention that these suckers can adhere in humid environment.

## Supporting Information

S1 Dataset The adsorption force data of different suckers. (XLSX)

S2 Dataset The texture analysis results of leech posterior suckers. (XLSX)

## References

[1] Aksak B, Murphy MP, Sitti M. Adhesion of biologically inspired vertical and angled polymer microfiber arrays. Langmuir. 2007; 23: 3322–3332. 17284057 10.1021/la062697t

[2] Asbeck AT, Kim S, Cutkosky MR, Provancher WR, Lanzetta M. Scaling Hard Vertical Surfaces with Compliant Microspine Arrays. The International Journal of Robotics Research. 2006; 25: 1165–1179.

[3] Menciassi A, Dario P. Bio-inspired solutions for locomotion in the gastrointestinal tract: background and perspectives. Philos Trans A Math Phys Eng Sci. 2003; 361: 2287–2298. 14599320 10.1098/rsta.2003.1255

[4] Stern-Tomlinson W, Nusbaum MP, Perez LE, Kristan WJ. A kinematic study of crawling behavior in the leech, Hirudo medicinalis. J Comp Physiol A. 1986;158: 593–603. 3723440 10.1007/BF00603803

[5] Tramacere F, Beccai L, Kuba M, Gozzi A, Bifone A, Mazzolai B. The morphology and adhesion mechanism of Octopus vulgaris suckers. PLOS One. 2013; 8: e65074 10.1371/journal.pone.0065074 23750233 PMC3672162

[6] Takashi M. Functional Morphology and Performance of Ecological Systems with Extreme Pressures: Waterfall Climbing and Predator- Prey Interaction in Amphidromous Gobioid Fishes. All Dissertations. 2013; 1120.

[7] Schliemann H, Goodman SM. A new study on the structure and function of the adhesive organs of the Old World sucker-footed bat (Myzopoda: Myzopodidae) of Madagascar, Verh. Naturwiss. Ver Hamburg (NF). 2011; 46: 313–330.

[8] Robert A, Peter Z. Therapeutic Use of Leeches: From the 'Annelids' or Medicine. University of Toronto Medical Journal. 2001); 79: 65–67.

[9] Teut M, Warning A. Leeches, phytotherapy and physiotherapy in osteo-arthrosis of the knee—a geriatric case study. Forsch Komplementmed. 2008; 15: 269–272. 10.1159/000158875 19001824

[10] Michalsen A, Moebus S, Spahn G, Esch T, Langhorst J, Dobos GJ. Leech therapy for symptomatic treatment of knee osteoarthritis: results and implications of a pilot study. Altern Ther Health Med. 2002; 8: 84–88. 12233807

[11] Orevi M, Eldor A, Giguzin I, Rigbi M. Jaw anatomy of the blood-sucking leeches, Hirudinea Limnatisnilotica and Hirudomedicinalis, and its relationship to their feeding habits. J Zool Lond. 2000; 250: 121–127.

[12] Qiao N, Bai Y, Wang G, Xu S. Comparative morphology study on the jaws of three leech species of the Genus whitmania Blanchard. Sichuan Journal of Zoology. 2015; 32: 526–529.

[13] Yang T. The specificity of chemoreceptors in blood-sucking leeches. Bulletin of Biology. 2001; 36: 8–9.

[14] He L. ESEM observation of some fresh biological samples. Journal of Chinese Electron Microscopy Society. 2003; 22: 669–670.

[15] Tang X, Dai S. ESEM observation of biological samples. Journal of Chinese Electron Microscopy Society. 2001; 20: 217–223.

[16] Stern-Tomlinson W, Nusbaum MP, Perez LE, Kristan WB. A kinematic study of crawling behavior in the leech, Hirudo medicinalis. Journal of Comparative Physiology A. 1986; 158: 593–603. 10.1007/BF00603803 3723440

[17] Busaba P, Arin N, Prasert S, Panat A. Morphology and histology of the adult Paramphistomum gracile Fischoeder, 1901. J. vet. Sci. 2013; 14: 425–432. 23820216 10.4142/jvs.2013.14.4.425 PMC3885736

[18] He B, Wu J, Chim S, Xu J, Kirk T. Microstructural analysis of collagen and elastin fibres in the kangaroo articular cartilage reveals a structural divergence depending on its local mechanical environment. Osteoarthritis and Cartilage. 2013; 21:237–245. 10.1016/j.joca.2012.10.008 23085561

[19] Tian J, Iwasaki T, Friesen W. Muscle function in animal movement: passive mechanical properties of leech muscle. Journal of Comparative Physiology. 2007; 193(12):1205–1224. 17987298 10.1007/s00359-007-0278-y

[20] Amend JR Jr, Brown E, Rodenberg N, Jaege H, Lipson H. A positive pressure universal gripper based on the jamming of granular material. IEEE Trans Robot. 2012; 28: 341–350.

